# Social regulation of emotion: messy layers

**DOI:** 10.3389/fpsyg.2013.00051

**Published:** 2013-02-15

**Authors:** Arvid Kappas

**Affiliations:** School of Humanities and Social Sciences, Jacobs University BremenBremen, Germany

**Keywords:** dynamic systems, complex systems, feedback, auto-regulation, cyberemotions

## Abstract

Emotions are evolved systems of intra- and interpersonal processes that are regulatory in nature, dealing mostly with issues of personal or social concern. They regulate social interaction and in extension, the social sphere. In turn, processes in the social sphere regulate emotions of individuals and groups. In other words, intrapersonal processes project in the interpersonal space, and inversely, interpersonal experiences deeply influence intrapersonal processes. Thus, I argue that the concepts of emotion generation and regulation should not be artificially separated. Similarly, interpersonal emotions should not be reduced to interacting systems of intraindividual processes. Instead, we can consider emotions at different social levels, ranging from dyads to large scale e-communities. The interaction between these levels is complex and does not only involve influences from one level to the next. In this sense the levels of emotion/regulation are messy and a challenge for empirical study. In this article, I discuss the concepts of emotions and regulation at different intra- and interpersonal levels. I extend the concept of auto-regulation of emotions (Kappas, 2008, 2011a,b) to social processes. Furthermore, I argue for the necessity of including mediated communication, particularly in cyberspace in contemporary models of emotion/regulation. Lastly, I suggest the use of concepts from systems dynamics and complex systems to tackle the challenge of the “messy layers.”

## Social regulation of emotion: messy layers

When I finished my PhD thesis on *control of emotion* (Kappas, 1989), the topic that is now commonly referred to as *emotion regulation* was considered somewhat peripheral to emotion science, but it clearly is not now (Tamir, 2011). Presently, there is much empirical research and, in consequence, a considerable number of publications on this subject. However, current views focus on emotion regulation as an intraindividual process. I will argue that there are benefits in reframing the concept of emotion regulation. Specifically, I will discuss emotions as multi-layered processes in which intraindividual processes are tightly coupled and often cannot be separated from interindividual processes. While the focus of emotion research arguably rests in the individual, I will briefly discuss the importance of embodiment and social interaction to prepare the key arguments of the present paper. I will argue that social layers are not only involved in the generation, as well as in the modulation/regulation of affective processes, but that social emotion generation and social emotion regulation can often not meaningfully be separated. This dynamic view has consequences on how we should think of emotion and emotion regulation, and how these should be studied. I will link the discussion of social emotion dynamics to the notion of auto-regulation that I have developed elsewhere (Kappas, 2008, 2011a,b) and also extend the range of social interaction to large numbers of individuals in cyberspace. I argue that emotions in this sense are self-regulating using social networks at different scales that provide not only empathic feedback but they direct the action of others in co-evolving social emotion cascades.

## Where is the emotion?

Most of us think of emotions as *intraindividual processes*. Evidently, emotions are subjectively very personal in nature. They relate to who we are and how we see and interact with the world. In fact, several theorists have pointed out a close link between emotions and the emergence of the self and/or self-consciousness (e.g., Damasio, 1999; Cabanac et al., 2009)—*I feel, therefore I am.* This view can also be traced to formal definitions of emotions by researchers in the area. Unfortunately, despite a long history of research, the definition of emotions is still a point of contention (Izard, 2010; Kappas, 2011a). However, arguably most current theorists, as lay-people do, consider emotions private processes. I will give a brief overview of different possibilities how to conceive of emotions, including not only as intra- but also interindividual processes. Some researchers have argued that emotions are social in nature (e.g., Parkinson, 1996), but I will attempt to push the envelope further by considering emotions also as properties of a dyad, a group, or a collective.

*Emotions* provide (1) responses to external and internal events, (2) help to anticipate situations in the future, (3) help to adapt to events in the past, and in consequence (4) afford an impetus to engage with the social and non-social world. Nevertheless, while these processes provide an interface to the world, feelings, physiological changes, and changes in action readiness rest subjectively inside of us. In current (western) thought, emotions are in fact located *inside of the brain*. This is, of course, consistent with how emotions are mostly studied in experimental psychology and neuroscience. Where else should emotions be? However, it is important here to remember that locating emotions exclusively in the brain is recent—and not, for example, seeing affect being a matter of the heart (e.g., Shweder, 1994)! As neuroscience shifts from its classical heritage in localizing faculties in specific brain areas (Uttal, 2001) to a more fruitful network paradigm (e.g., Bressler and Menon, 2010), the question emerges how large should the (bodily) networks be that we consider relevant for emotions? If the gut really plays a role in the *gut feeling*, should the gut then not be part of the network?

I argue that it may often be a useful analytical convention to locate emotions in the brain, but that at times it can be helpful to consider different perspectives. There is clear evidence that peripheral processes play an important part in cognition, motivation, and emotion—and this includes not only the nervous system, but also the endocrine system (e.g., Maier and Watkins, 1998). For the purpose of presenting these two ways of conceiving of the intrapersonal location of emotions, I will use the terms *centralist* (in the sense of brain-based) vs. *peripheralist* (in the sense of all-body) model. Note that both terms are not used consistently in the literature. The origin of the current *centralist view* of emotion can be anchored in the position of different theorists, particularly in the 19th and 20th century (see also Gendron and Barrett, 2009), but is still very much dominant, for example in current appraisal theories (see, Scherer et al., 2001; Ellsworth and Scherer, 2003) and neuroscientific approaches to appraisal theory (e.g., Ochsner et al., 2002). Peripheralist views are frequently associated with notions elaborated by William James. In his view (e.g., 1884), there could not be a normal *feeling*, or in current terminology, *subjective experience of emotion*, without the perception of bodily responses playing an important part (see also Frijda, 2009). In other words, according to this view, peripheral responses are extremely important to the *subjective component*—the feeling (Reisenzein et al., 1995; Dunn et al., 2010). This is notwithstanding that the typical route of emotion generation may pass from (1) external stimuli to (2) a perceptual process, to (3) the elicitation of behaviors, and peripheral responses, back to (4) perception of these changes (Ellsworth, 1994, but also Reisenzein et al., 1995). The body, according to this theory, is part of a regulatory loop that cannot be taken out of the experience of emotion. Today, interest in the peripheralist view is associated with three lines of research, namely (1) studies related to the facial-feedback hypothesis (see below), (2) Damasio's somatic marker hypothesis (e.g., 1999), and (3) the wide-spread recent interest in embodied processes, not only in emotion, but also in motivation and cognition have forcefully revived the idea that the body has an important part to play in emotion (Barsalou et al., 2003). In other words, while the brain might be necessary for all mental processes it often is not sufficient to explain all of, cognition, motivation, and emotion. Hence, these can be conceived of as intraindividual processes that are *inside of me-the-body*, and not just *inside of me-the-brain*. This is not only a question of phenomenology, but refers to numerous studies manipulating bodily posture, facial patterns, or temperature to impact emotion-related processes, frequently outside of participants' awareness.

While there are conditions where peripheral bodily processes are neither necessary nor sufficient for changes in phenomenology (see Reisenzein and Döring, 2009), this does not render the notion of emotion as bodily processes as useless, as there are many situations where they are (see Ohira, 2010). Furthermore, there are also affective behaviors or physiological changes that can be observed objectively apart from subjects' phenomenological awareness (e.g., Winkielman and Schooler, 2008). In summary, there is no question that brain activity is crucial for all affective processes. However, it is relevant for how we conceive of emotions (and emotion regulation) whether a model/theory should include the body as an integral part of or simply provide an entry point to (“this is where bodily feedback arrives”) emotion. I argue that sufficient evidence has accumulated over the last decades that peripheral processes modulate, if not jump-start affective processes at times. Because there is a complex interplay of afferent and efferent pathways between the brain and the periphery—even the endocrine system (Maier and Watkins, 1998), it would appear, from a systems dynamics point of view, problematic to reduce these nested feedback loops within the body to a mere add-on. There are dynamic properties that relate to the physical representation of processes in the periphery that require the body to be an integral part of any emotion model. I will now consider whether one can conceive of emotion as an interindividual process.

## The social nature of emotion

### Darwin's contribution to the concept of social functions of displays and feelings

Central to any discussion on the social nature of emotion is expressive behavior. A minimal consensus holds that emotions *may* be accompanied by expressive behavior and that expressions are often universally interpreted as signs of emotions (Russell, 1995; Kappas, 2003; Kappas et al., 2013). Some researchers hold much stronger views regarding the relationship of expression, feeling, and physiological activity—such as that there are innate links between affect programs and specific expression patterns and in turn the perception and interpretation of expressive behavior (see Russell and Fernández-Dols, 1997). This notion is arguably also a consequence of the history of emotion science.

Most current researchers would agree that a seminal point in the scientific study of emotions was the publication of Darwin's *The Expression of the Emotions in Man and Animals* (1872). It is noteworthy, that Darwin's in-depth discussion of origins and functions of expressive behavior was at the center of the birth of modern emotion research (see also Cornelius, 1996). In other words, the expression offered a different and more concrete approach to emotions than more abstract discussions of feelings or motivations that might have characterized much of the philosophical treatment of emotions pre-Darwin. Arguably, Darwin's observations and arguments have shaped much of the research and theory in the time since. While expressing and perceiving emotions seems to emphasize the social nature of emotions, curiously enough, Darwin's focus was *not* on interindividual processes. Instead, he spent many pages to explain how specific expressions could have evolved as a consequence of other (non-social) functional origins, such as regulating information inflow, respiration, or the intake of food. Only toward the end of the *Expression*, and frequently overlooked, Darwin mentioned two important ideas regarding the importance of expressions in the here-and-now. One refers to the social nature of expressions and the second to intrapersonal emotion-regulation effects of controlling expressive behavior.

Since the publication of the *Expression*, Darwin has been invoked many times as a key emotion theorist. For example, the work of Paul Ekman and his collaborators in the late 1960's of the 20th century focused on the universality of certain expressions, inspired by Darwin. Using some much-cited experimental evidence (Ekman et al., 1969) they suggested that, for a limited number of emotions, strong universals in the expression and perception exist. Cultural differences that can be observed are, according to Ekman and Friesen (1969), due to learned *display rules*. While some of the inferences of these researchers are contested (see Russell and Fernández-Dols, 1997), there seems to be a widely shared consensus nowadays that there are biological constraints that are linked to certain facial movements that over the course of intrapersonal development are used and interpreted in the context of idiosyncratic as well as culturally shared norms and expectations (Scherer and Brosch, 2009; Averill, 2012; Boiger and Mesquita, 2012, see also Fogel et al., 1992). In other words, in this view emotional displays serve communicative purposes and social contexts have a modulatory function. But the emphasis is still on emotion as an intrapersonal process. This is also the view that is predominant in much of the emotion regulation literature (see below).

In the 1990's, an important reinterpretation of Darwin's view on “expressive behavior” was suggested. Fridlund (1991, 1994) argued that Darwin had not suggested that “expressive” behavior was primarily linked to emotion, but to social goals instead. In other words, expressive behavior, according to this view, is not a readout of emotions, but in the shape of *emotional displays* serves specific purposes in interaction. Fridlund's original research has inspired further studies. The current reading of the empirical data available suggests that facial behavior is associated with emotion as well as with social goals (Hess et al., 1995; see also Jakobs et al., 1999a,b, 2001; Parkinson, 2005)—moderated by the actual or only implied presence of conspecifics and their social relationships. Arguably, the notion of display rules is still relevant (see Matsumoto, 2009), but it is not sufficient to explain the low cohesion between what people feel, what they show, and their physiological activation (e.g., Mauss and Robinson, 2009). Nevertheless, few researchers focus on what emotional expressions *do* in social context (Parkinson, 1996, 2005). Instead, in my reading, most empirical studies on facial behavior today are interested in using expressions as a diagnostic tool to replace self-report of feeling states. In contrast, particularly in sociology there is the notion that emotions serve to bind individuals together (von Scheve and von Luede, 2005) and that individual emotions may mark relationships (Kemper, 2011). In these contexts expressive behavior is part of the mechanisms that achieve these interindividual goals. How can expressive behavior achieve such functions?

### The development of emotions in face-to-face and mediated social interaction

Darwin already hinted at the possibility of a joint process of emotion elicitation in the interaction of mother and infant. I have previously referred to such preverbal dyadic evaluation and action processes as *distributed affective processing* (Kappas and Descôteaux, 2003) and suggested that several appraisal dimensions could be affected by manifest or imagined social context (Kappas, 1996). For example, a child may not have enough information to appraise whether an event or object is beneficial or harmful and thus, the appraisal is “outsourced” to mother by querying her expression. In other words, the cognitive process involved in dealing with a situation here transcends an individual brain. This mechanism involves the externalization and perception of expressive behavior that forms a bond in the face of a particular affordance—dealing with an unclear situation. Rochat and Striano (1999) have referred to such ontologically early social exchanges as the *cradle of social cognition*.

As the child grows up, there is much to learn regarding how to evaluate particular situations or events in order to thrive, adapt, and in the extreme, survive (see Fogel et al., 1992). There are apparently some types of stimuli that elicit universal behavioral responses (such as objects approaching us with very high speed) or attract attention (such as faces; see Kappas and Olk, 2008). Similarly, some types of stimuli might be easily associated with particular meanings, such as snakes or spiders when paired with affective expressions of others (e.g., Öhman and Mineka, 2001). However, the majority of affect-knowledge in humans will not be linked to direct personal experience with an eliciting situation, but to repeated and frequent exchanges with conspecifics, such as care-givers, friends, and others where relevant information is shared. Social sharing of emotions is prevalent in all cultures and plays an important part in managing relations throughout life as well as in distributing emotion-related knowledge (e.g., Rimé, 2009). Culture shapes these social processes in many ways. Specifically, culture affects how to appraise a particular event or may even impact what constitutes an emotion (see Shweder, 1994; Cornelius, 1996; Mesquita and Leu, 2007). Furthermore, differences in language impact how a particular syndrome of affective processes might be referred to (Wierzbicka, 2009; Kagan, 2010). Thus, a name for a particular script might be available in one language, but not the other (e.g., *Schadenfreude* in German or *amae* 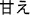 in Japanese). There is shared knowledge regarding when to feel a particular emotion or not (see Hochschild, 1983), or which emotions might be desirable *in a particular culture* (Mesquita and Ellsworth, 2001; von Scheve, 2012). All of these are relevant to social regulation of emotion.

Several contemporary authors argue that in humans the majority of emotional episodes typically occur in the context of social interactions (see Parkinson, 2011; Boiger and Mesquita, 2012). This relates to actual interactions, implicit interactions (e.g., Fridlund, 1991; Hess et al., 1995), or even when there is a perceived lack of interactions (Cacioppo and Patrick, 2008). Of particular importance for the study of these phenomena is the distinction between the flow of affective cascades occurring in real time and the epigenetic development and shaping of emotional rules and norms (see also Fogel et al., 1992; Campos et al., 2004; Boiger and Mesquita, 2012). Yet, both types of processes underscore the importance of emotions as social processes. The social shaping of emotions clearly is not only a function of experiences in the here-and-now, but also part of the sharing of emotions, as mentioned above in a personal context. However, social influences go much further than person-to-person processes. In contemporary societies, we have to consider also mediated, and specifically mass-mediated contexts.

Recent developments in mass communication have a strong influence on normative processes relevant for the elicitation and the expression of emotional episodes (Bandura, 2009). Emotion portrayals and prototypical scripts of when a particular emotion might be appropriate or not have been shared via printed media for hundreds of years (see Mar et al., 2008; Hogan, 2010). Going back further, in the context of theatre and oral history there is an influence in the scale of thousands of years. However, it is the development of film and television that has lead to an explosion of the sharing of informative and normative material regarding emotions in the 20th century (see also cultivation analysis; e.g., Morgan et al., 2009). In this context, there are media that are frequently national (e.g., television: soap operas, tele-novelas, and film) as well as global, such as large-scale movie productions (which can also be distributed on TV and in cyberspace, but that originate in the movie format and are originally distributed for presentation in movie theatres). What is being communicated ranges from concrete observable responses (e.g., Carroll and Russell, 1997) to specific situations, to complex scripts and narratives that include issues such as if A does X_1_ and B does X_2_ then A might feel Y_1_ which is expressed as pattern Z_1_ in the presence of B, C, and D, but pattern Z_2_ in the presence of E … etc. Just as the infant is engaging with the physical environment in practicing and building up categories of cause and effect loops, from childhood, frequent and repeated exposure to stories (socially shared, literature, film, TV) creates and reinforces knowledge structures regarding social interactions and particularly emotions which are at the heart of these processes. As opposed to literature where much imagination is required to fill in the blanks, we are now exposed to thousands of emotional expressions associated with specific elicitors, in specific social situations in mass media. The impact of mass-mediatized socialization of emotion rules and norms is likely rather under-than overestimated. In a way this is also a social regulation of emotions by defining the expectations and the stereotypes, but I will not focus on these slow processes in the present context.

The recent development of cyberspace (Boyd and Ellison, 2007) has complicated matters further by blending the boundaries between interpersonal communication and mass-communication. For example, in the context of blogs, individuals may share their emotional experiences with a large audience and may or may not include the possibility to respond with comments or other means, such as email, or messaging systems (e.g., Thelwall and Wilkinson, 2010). In the context of forums, exchange is part of the design, but many visitors to a forum might *lurk* without ever contributing. While much of the research on how emotions are communicated online is so far based on text-based computer mediated communication, there is an increasing amount of multimodal content, including the possibility for face-to-face communication in cyberspace (Kappas and Krämer, 2011). It is only very recently that large-scale research has started to investigate the exchange of emotions over thousands or even millions of online posts (e.g., Chmiel et al., 2011).

The rapid increase in cyberspace communication, specifically as regards the social web, is not only a technological phenomenon. Instead, the motivation to be socially connected interacts with the affordances of the Internet in rapidly evolving ways. Within few years, particular media come and go (e.g., MySpace, Facebook, Twitter), as they are differentially successful in catering to users' social needs and desires. However, regardless of the features of specific media, for example regarding modalities, speed, or anonymity, it is the online sharing of emotions (Rimé, 2009) that is common and central. Indeed, the prevalence of emotional terms online is considerable (Harris and Kamvar, 2009). There is no evidence that online exchanges are less emotional than online interactions (Derks et al., 2008). Thus, it appears imperative to include cyberspace in any contemporary discussion of the social nature of emotions, even if there are still local differences in Internet availability and usage. The complex tapestry of connecting with people that are not only geographically, but also culturally heterogeneous, is bound to affect local and personal norms in this and the coming decades. These developments relate to aspects of the generation, and as we will see later, the regulation of emotions.

In summary, there are numerous ways in which emotions are social: (1) the situations in which emotions are elicited are frequently social, (2) the contents of the events eliciting emotions are frequently social, (3) the acquisition, and shaping of rules and norms are largely social, (4) sharing of emotions is driven by social needs and serves a variety of social functions. Furthermore, (5) deficits in emotion expression or interpretation lead to social problems (e.g., if particular expressions cannot be perceived due to reduced vision or hearing; if particular expressions cannot be produced due to conditions such as Möbius Syndrome, or Parkinson's Disease; or if attention to and interpretation of non-verbal behaviors is challenged, such as in the context of Autism Spectrum Disorder).

Psychologists tend to slice the world into packages the size of individuals and occasionally see aggregates of *n* > 1 as simply the sum of individuals. This view of course ignores that there are processes that occur only (1) in actual or implied *interaction* and (2) due to the interaction of *specific individuals*, and that (3) in *specific contexts* (see also the principle of non-additive determinism in the context of multilevel analyses: e.g., Cacioppo and Decety, 2011). Popular concepts, such as an *angry mob*, as vague as they may be, reflect that there seems to be a conventional notion of *collective emotions*. At a scientific level, there has been some interest in recent years in understanding collective emotions. This does not only refer to theoretical advances (Huebner, 2011), but also attempts to tackle the complexity of empirical approaches to collective emotions (Schweitzer and Garcia, 2010). One area where there exists some systematic research comes from the workplace context. Here, for example, the concept of *emotional climate* is particularly relevant (e.g., Yurtsever and De Rivera, 2010). There might also be a climate in cyberspace which could be characterized by specific language use (Thelwall et al., 2010) reflecting implicit norms, or the dynamics with which emotional statements elicit emotions in subsequent emotions (e.g., Chmiel et al., 2011). The latter is particularly interesting because online communities are often much more fluid with regard to who is a member and for how long (Trier and Bobrik, 2009). If indeed there exist emotional norms that evolve in this context, this may be relevant for a better understanding of how emotion norms develop and are communicated, because there are particular affordances for online research of emotions. For example, large bodies of data are already available for research. In turn, this begs the questions how norms migrate from place to place and or shape norms and behaviors in individuals.

### Interpersonal emotions

Above I argued that we could think of instances, where the emotion process should be located in the body as a whole. Can we conceive of instances where the emotion process should be considered as being truly interpersonal (Huebner, 2011)? I have already touched upon the concept of emotional climate—this seems to be a property of an institution which is instantiated in the brains of people—where the probability of emotions is skewed as a function of habitual interactions and norms. The same people might act differently in a different context—much in the way Goffman conceived of as roles. However, there is clearly something that exists only in the context of the institution.

At a smaller scale we might think of a small group, such as a family, where emotions are organized in the dynamic exchange between the members, or at a dyadic level there exist concepts such as attachment, or couples that clearly hint at a reality of emerging properties of aggregates of individuals in different relationships. The last decades of social psychology have been characterized by studying emotions in social isolation—this is due to two factors, (1) the dominant view of emotion regulation considers responses in social isolation to be genuine and untainted, and (2) the move from social psychology to social cognition has focused on implicit processes that can easily be studied in individuals in isolation. Increasingly there are calls for paradigms that study emotions in actual social interactions (e.g., Fischer and van Kleef, 2010). It is not enough to discover that the brain is social—we also need to study the brain in social contexts. The aforementioned research in the context of behavioral ecology (e.g., Fridlund, 1991) is a good case in point. It *does* make a difference whether someone is present, and it matters whether this is a stranger, or a friend (Hess et al., 1995). Why is the social context in the experiment so rarely considered? Even when using brain imaging, it is possible to manipulate the actual social situation, such as the work of Jim Coan and his colleagues on responses to pain when the hand of the person is held by the partner, a stranger or nobody—while the participant's brain is being scanned (Coan et al., 2006; see also Coan, 2011).

We are currently conducting experiments in my laboratory and in collaboration studying collective emotions in cyberspace (see www.cyberemotions.eu). In some of the recent experiments we had participants communicate in real time via a computer. They were physically separated, but online connected via text-based computer-mediated-communication. In one condition of one of the experiments we asked participants to get acquainted with each other, in the other we did not—this manipulation changed not only subjective experience, but also expression and electrodermal activation (Kappas et al., 2012) in these interactions. These are processes that emerge in real time and they apparently scale to e-communities of considerable size (Chmiel et al., 2011).

**Figure 1:**
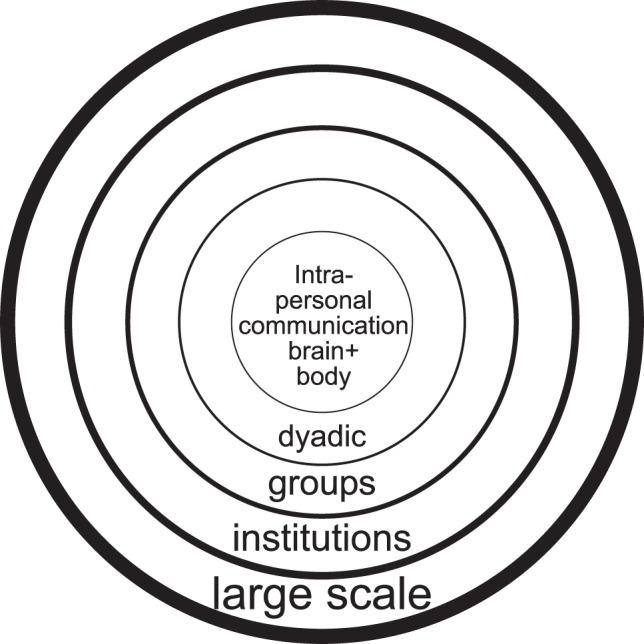
**“Onion layer” model suggesting emotions occurring at multiple nested levels.** However, while the image suggests a clean hierarchical structure, in reality non-adjacent layers interact in complex ways in the generation (and regulation) of affective processes. Thus a “messy layer” model would be more appropriate as is argued later in the paper.

I suggest that we think of emotion generation and modulation (below) at different levels. In this case, the emotion can be a property of a dyad, a group, or the individual (see Figure 1). I believe there is an added advantage in not reducing our concept of emotion to whatever information enters a single brain and conceive of social interactions as the sum of individual processes. The word interaction is key here, as different levels of emotion generation (and regulation) are likely to act (1) concurrently and (2) in interaction (see also Butler and Gross, 2009). The work of John Cacioppo and his colleagues of conceptualizing multi-level analysis of psychological processes and behavior is particularly helpful here (e.g., Cacioppo et al., 2000). Nevertheless, I want to emphasize that the argument I am making here is to not look (only) at the emotions of individuals at different levels of social aggregation, but to actually consider emotions of a group, a company, or a dyad. This does not mean that all individuals perceive or express their emotions identically, but that there is a residual level at which the emotional processes that can be observed exist in the interaction. Consider the metaphor of a play. When you are watching a play, the play has a reality of its own and is not just a place in time and space where several actors give individual performances. A symphony is a score and complex interactions between musicians and conductor and audience, not a bunch of musicians acting out something in relative synchrony. While this might be a correct statement, it does not provide the same usefulness as the acknowledgement that the immaterial piece of music posesses a certain reality that transcends individuals.

## Regulation of emotions

Not much space needs to be dedicated here regarding what is currently thought of as *intrapersonal regulation* of emotions, due to the many recent publications on the issue, most of them authored or edited by James Gross and/or his collaborators (Gross, 2007; see also Vandekerckhove et al., 2008). Emotion regulation in this school of thought focuses primarily on (often) voluntary efforts to change ongoing or expected emotional episodes of individuals via effortful cognitive and expressive processes—“the process by which individuals influence which emotions they have, when they have them, and how they experience and express these emotions” (Gross, 1998, p. 275).

While emotion regulation is seen as a recent topic, most of the current empirical work in individuals goes straight back to Lazarus' notion of and empirical research on appraisal and reappraisal from the early 1960's (e.g., Lazarus and Alfert, 1964; Lazarus et al., 1970; Lazarus and Folkman, 1984) and Darwin's suggestions regarding feedback processes (Kappas, 1989) in the 19th century. With regard to expressive feedback Darwin wrote in the conclusion of *Expression*:

The free expression by outward signs of an emotion intensifies it. On the other hand, the repression, as far as this is possible, of all outward signs softens our emotions. He who gives way to violent gestures will increase his rage; he who does not control the signs of fear will experience fear in a greater degree; and he who remains passive when overwhelmed with grief loses his best chance of recovering elasticity of mind. These results follow partly from the intimate relation which exists between almost all the emotions and their outward manifestations; and partly from the direct influence of exertion on the heart, and consequently on the brain. Even the simulation of an emotion tends to arouse it in our minds (1872, p. 366).

This is a rather remarkable list of proposals that foreshadow much of what current research on feedback and embodiment suggests. Initially (e.g., Tourangeau and Ellsworth, 1979), there was some skepticism regarding research suggesting an impact of expressive behavior on emotions, specifically the so called *Facial Feedback Hypothesis*. However, later reviews were more positive that feedback effects from expression on subjective experience and physiology (Adelman and Zajonc, 1989; McIntosh, 1996) exists and can be reliably demonstrated empirically. These findings are now discussed in a larger context of embodied emotion/motivational processes, where facial and bodily movements interact with affective processing (e.g., Niedenthal, 2007; Price and Harmon-Jones, 2010). There is no doubt that Darwin held already the view that volitional modulation of emotion components—particularly expression, would lead to modulation of the other components. This view is often erroneously dated later, for example, linked to William James or other authors later in the 20th century.

Particular interest in recent years regarded how automatic emotion regulation processes might be (e.g., Mauss et al., 2007; Koole and Rothermund, 2011) and to what degree emotion generation and emotion regulation can be distinguished (Gross et al., 2011a). I have argued in the past that these often cannot be distinguished (Kappas, 2008) and specifically in discussing the case of *auto-regulation* in the case of negative emotions (Kappas, 2011a,b) where the actions motivated by the emotion lead to its own termination by modifying the eliciting situation. Given that emotions typically involve a strong motivational component that involves modulation of the emotional state itself (decrease, increase, change, prolong the current state), this is not surprising. Whether or not a scientist wants to grant emotions the power to auto-regulate depends on how thin one slices the situation that is under study.

Consider the following scenario: a parent tells the child that it is already late and that it would be time to go to bed. The child starts to cry. The parent gives in and postpones bed time by 15 min. The child stops sobbing and smiles through the tears. For the sake of avoiding conceptual discussions about where the emotion here might be, I will frame this scenario for three different types of readers in three theoretical contexts: (1) having to go to bed violates the goals of the child, it feels that it cannot influence the situation and it starts to cry because it is sad (appraisal and basic emotions view). (2) Understanding that the end of play time is at hand, the child is frustrated and cries. Based on the context and the interpretation of the situation, the parent, and possibly the child view this as an episode of sadness (for a modern constructivist view see Barrett, 2011). (3) The desire to stay up leads (consciously or unconsciously) to the strong social motivation to change the parent's mind (behavioral ecology). However, you slice it—the behavior of the adult triggered an emotion/motivational process in the child in the course of which the behaviors of the child lead the adult to change the rules again, which in turn modulates the emotion/motivational process. In other words, the emotion auto-regulates itself by generating and modulating behaviors in both participants of the interaction. To me this is an example of auto-regulation—and it also nicely segues into the section how emotion regulation is often social and does not often permit to distinguish generation and regulation processes.

To be clear, I do not argue that all instances of emotion regulation could be reduced to auto-regulation. Instead, I hold that in many instances auto-regulation serves to terminate or modify the eliciting situation to self-terminate the emotion. In the case of pleasant states—positive emotions—there is a tendency to maintain or increase aspects of the situation to maximize pleasure. The voluntary regulation via cognitive or behavioral routes is the exception if auto-regulation fails. For example, if going to the dentist induces anxiety then avoiding the dentist auto-regulates the anxiety in the moment. However, because this is dysfunctional in the long-term voluntary emotion regulation strategies are employed to follow through with the anxiogenic situation, unless the fear is too intense. This is a case where auto-regulation does not help the goals of the individual, but I believe that these situations are less frequent then typically held in the literature and that emotions take care of themselves, metaphorically speaking.

## The social regulation of emotions

The neurocultural theory proposed by Ekman and his colleagues is particularly elegant in that it accounts for biological universals as well as cultural variants. While cultural rules modulating expressions have been discussed already in the 19th century by theorists such as Wundt, and indeed Darwin, it is the concept of “display rules” (Ekman and Friesen, 1969) that has captured the imagination of many researchers in the field of emotion—even appraisal researchers.

I have argued earlier (e.g., Kappas and Descôteaux, 2003) that practical reasons would suggest that emotion regulation should be part of emotion theories and not a tool for *post-hoc* explanation of inconsistent findings. For example, if a given theory predicts that individual A, confronted with Situation S should show behavior X_1_ then this should allow a simple empirical test of the theory: bring person A in Situation S, measure behavior, and if I find X_2_ instead of X_1_, then the prediction was wrong. The theory might need revision. However, this is not what happens frequently in emotion research apparently. All too often behavior X_2_ is explained ex post facto as the consequence of variability in appraising S (appraisal theory), or X_2_ being the consequence of display rules (e.g., neurocultural theory), or both. I argue that if (1) display rules are potentially interfering with displays, and (2) a theory is primarily regarding the relationship of displays, feelings, and other emotional components, then (3) the rules must be part of what needs to be modeled. Note, that the notion of display rules is an instance of social regulation of a component of emotion that is present in *all* social situations. In other words, there seems to be a large agreement that there are social rules governing displays in all social situations!

If Fridlund (1994) is right, then there is *always* a social context that influences expressive behavior, even if humans are physically alone. This would mean that one cannot interpret any display without taking social regulation into account. There is no expressive behavior that is not affected by social regulation. Furthermore, if *feeling rules* exist (Hochschild, 1979, 1983), then we are almost constantly affected by beliefs of what is proper and what is not. In fact, Scherer (2001) has embedded a comparison with social and own norms into his popular *Component Process Model* of emotion. According to this theory, every event, every situation, is also evaluated with regard to social norms. Taking these theoretical approaches seriously implies that the social aspect cannot be divorced from studying *any* emotion generation (see also Parkinson, 1996).

**Figure 2:**
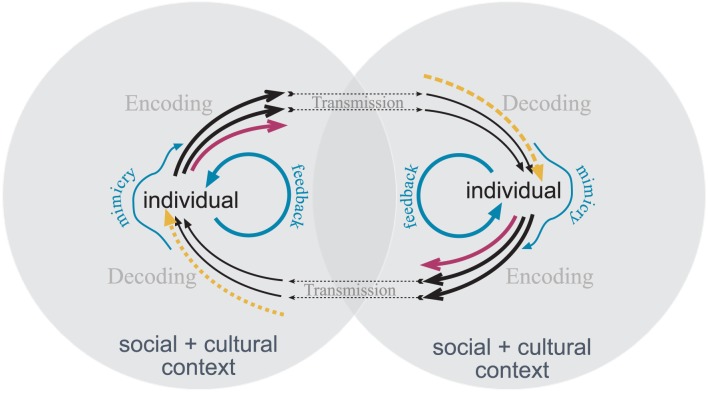
**Superlens model adapted from Kappas (1991) and Kappas and Descôteaux (2003).** Communication pathways are indicated in black, expressive regulatory pathways in blue. Red arrows indicate objectively measurable displays that are not transmitted/received. Yellow arrows indicate erroneously perceived displays, for example due to stereotypes and expectations. Encoding and decoding of affective/motivational displays are a function of individuals' social and cultural context that are part of every individual. The intersection symbolizes the situationally shared context including all aspects that affect transmission, such as distance or noise.

If regulating displays, be they facial, vocal, or postural, impacts subjective experience and physiology, then automatic or effortful regulation in the sense of *display rules* will also affect other emotional components. For example, if a culture holds that boys do not cry then this will via feedback processes impact feeling and physiological arousal. This is why it is important to conceive of emotions as embodied processes. Even if the effect sizes of such influences might be small, they might tip systems to go into particular states if they are not at a steady state. How can the social regulation then be denied to be part and parcel of all affective processes? I have proposed a *Superlens Model of Communication* (1991; Kappas and Descôteaux, 2003) that takes imitation and mimicry into account (see Figure 2).

If there are mimicry and imitation processes in interaction (regardless of the role of mirror neurons; see Decety, 2010; also Parkinson, 2011) and these in turn affect how we feel—again, it is not conceivable to imagine emotional processes in interaction that would not be potentially affected by social processes. To me it is baffling how one could even conceive of “clean” or “untainted” emotional processes in the laboratory, or in the real world. Based on the notion of implicit sociality we can assume that even participants who are physically alone in an experiment are subject to social influences and like a *free monadic radical* will link to whatever social synapse that is available, e.g., the representation of the experimenter. Similarly, there is no situation in which an individual could be non-cultural. Instead, there are likely situational features that can prime and shape how culture affects mental processes and behavior. But we are always embedded in a cultural and social context.

**Figure 3:**
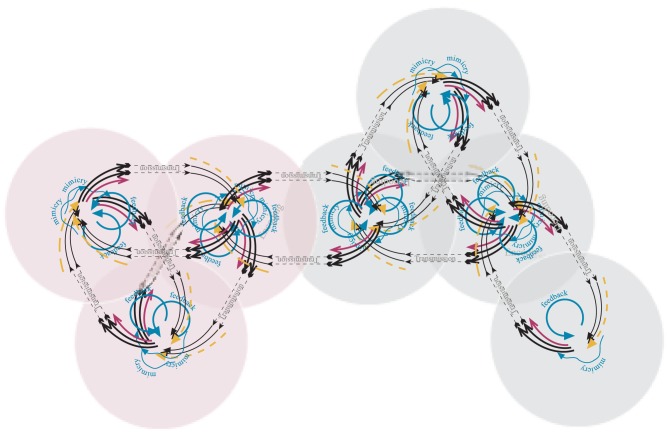
**Hyperlenses.** Seven hypothetical individuals interact in a communication structure that involves several dyads and triads—for example in the context of a party. The color of the context circle suggests here two different groups participating. Hence, some interactants share a more similar context than others. The meaning of all arrows is analogous to that in Figure 2.

In many settings more complex interaction topologies exist. Figure 3 shows a hypothetical scenario where seven individuals interact.

The social regulation of emotions is a messy affair to analyze. It is all too easy to capitulate and argue that there is, speaking exaggeratedly, simply no sense in considering everything that ever happened as a cause of emotion and regulation. Of course not. This sounds like a reasonable comment. However, the question is where one should delineate the boundaries of episodes in a way that we can really describe, explain, and possibly intervene in real life situations. Layers are messy because they are *not* organized like an onion. Different layers of emotions and different layers of regulation interact in complex ways—whether this is the impact of cultural display rules on intraindividual regulatory processes via facial feedback or the fact that family-idiosyncratic use of facial gestures interacts with cultural rules when visiting another country. Nowhere perhaps is the situation as complex as when interpersonal communication and mass-communication intermingle in cyberspace. Here e-communities come into being, develop new rules of (n)etiquette that are constantly in flux and that cause easily miscommunication. Sudden flaming wars can easily erupt based on the subjective experience of being insulted without any bad intention. In the past, such misunderstandings would be considered a case of codes that are not shared between all participants. However, considering the dynamic unfolding of the (emotional) exchanges online can be seen as an instance of a complex, multipersonal interaction with different goals on the one hand, and different effects on the other. Certainly, there is much to do here, because of the ubiquity of mediated communication that is rather increasing than decreasing in years to come.

The notion of auto-regulation holds that the “regulation” of emotion is part of the brief of itself in a rather recursive manner. Social regulation is one important facet of auto-regulation in that expressive behavior not only informs others, it *moves* them to do something, it *biases* their decisions, in this sense, negative emotions can impact others with the consequence of these emotions being terminated, as in getting support, or positive emotional states can impact others to reinforce themselves, as in amusement or desire. In this sense we are always embedded in social networks with different life-cycles (from the life-time of a family, to the brief minutes of a shared bus ride) where emotions are generated, moderated, regenerated, terminated, or reinforced as a function of how individuals affect each other in socio-cultural fields. It is because of this, that social layers should not (and cannot) be ignored in emotion research and that if the function of emotions involve their own regulation then generation and regulation of emotion are difficult to separate. Scenarios such as this appear messy because they do not easily lend themselves to the isolation of causes and effects that clean experimentation demands. However, they might help to understand the limitations of much of present emotion research, such as the challenges of low coherence between emotion components and the relationship of phenomena studied in the laboratory and those observed in the real world.

## Summary

I argue that there are many reasons to consider emotions not only a property of individual brains or bodies but of couples, families, cliques, teams, clubs, parties, companies, or e-communities. In my mind, this is one way in which emotions can be social. To study these types of emotions it is useful to take an interdisciplinary approach and collaborate with disciplines that naturally deal not with individuals, but with larger aggregates (von Scheve and von Luede, 2005).

In this view, it is natural that social forces are generative *and* modulating—in other words, the elicitation and the regulation of emotions are difficult to separate (see also Gross and Barrett, 2011). I have made this argument before in the context of individual emotions (e.g., Kappas, 2008, 2011a,b), and I extend it here to emotions of social entities. Typical counter arguments involve cases where generation and regulation can be (somewhat) cleanly separated (Gross et al., 2011b). However, I do not argue that *all* instances of emotion that we observe, whatever the mix of dependent measures is used, must be clearly linked to regulation. What I do point out, is that there are many instances in the classical, individual centered approach, as well as looking at more complex social structures (Rimé, 2009), where regulation and elicitation can best be described by nested layers of feedback loops. Because of this, theories of emotion should include these layers of regulation to permit the type of empirical testing that is necessary for a theory to be scientific. If any emotion theory is leaving regulation outside of their scope, there is no possibility to conduct proper tests regarding its validity. This calls for more real interaction in emotion studies (Rimé, 2009; Fischer and van Kleef, 2010). My colleagues and I have tried to achieve this either by manipulating social context in the laboratory, or by branching out into cyberspace and trying to assess the emotional behavior of large aggregates of individuals in e-communities (Chmiel et al., 2011).

Dealing with nested layers is messy because all layers can potentially influence emotional components (e.g., facial muscle activation). Research programs are required that can attempt to disambiguate the interaction of these layers. On the one hand, in the context of social neuroscience, there is much discussion of how to deal with the mutual interrelations of different layers (e.g., Cacioppo and Decety, 2011). On the other hand, as we start to deal with networks of people, we also need different ideas how to deal with these dynamic systems, and this calls for the science of complex systems (e.g., Chmiel et al., 2011; Garas et al., 2012). Combining these two approaches might be particularly fruitful in disambiguating the messy layers of emotion and emotion regulation.

### Conflict of interest statement

The author declares that the research was conducted in the absence of any commercial or financial relationships that could be construed as a potential conflict of interest.
